# CaARP1/CaSGT1 Module Regulates Vegetative Growth and Defense Response of Pepper Plants against *Phytophthora capsici*

**DOI:** 10.3390/plants13202849

**Published:** 2024-10-11

**Authors:** Xia Li, Yahong Weng, Yufeng Chen, Kaisheng Liu, Yanyan Liu, Kan Zhang, Lanping Shi, Shuilin He, Zhiqin Liu

**Affiliations:** 1 Key Laboratory of Ministry of Education for Genetics, Breeding and Multiple Utilization of Crops, Fujian Agriculture and Forestry University, Fuzhou 350002, China; 2 College of Agriculture, Fujian Agriculture and Forestry University, Fuzhou 350002, China; 3 Jiangsu Coastal Area Institute of Agricultural Sciences, Yancheng 224002, China

**Keywords:** CaSGT1, CaARP1, *Phytophthora capsici*, defense response, vegetable growth

## Abstract

Pepper (*Capsicum annuum* L.) suffers severe quality and yield loss from oomycete diseases caused by *Phytophthora capsici*. CaSGT1 was previously determined to positively regulate the immune response of pepper plants against *P. capsici*, but by which mechanism remains elusive. In the present study, the potential interacting proteins of CaSGT1 were isolated from pepper using a yeast two-hybrid system, among which CaARP1 was determined to interact with CaSGT1 via bimolecular fluorescence complementation (BiFC) and microscale thermophoresis (MST) assays. CaARP1 belongs to the auxin-repressed protein family, which is well-known to function in modulating plant growth. The transcriptional and protein levels of CaARP1 were both significantly induced by infection with *P. capsici*. Silencing of *CaARP1* promotes the vegetative growth of pepper plants and attenuates its disease resistance to *P. capsici*, as well as compromising the hypersensitive response-like cell death in pepper leaves induced by PcINF1, a well-characterized typical PAMP from *P. capsici*. Chitin-induced transient expression of *CaARP1* in pepper leaves enhanced its disease resistance to *P. capsici*, which is amplified by *CaSGT1* co-expression as a positive regulator. Taken together, our result revealed that CaARP1 plays a dual role in the pepper, negatively regulating the vegetative growth and positively regulating plant immunity against *P. capsici* in a manner associated with CaSGT1.

## 1. Introduction

Pepper is one of the most economically valuable vegetables worldwide; however, it is also highly susceptible to oomycete diseases, particularly those caused by *Phytophthora capsici* infection. This pathogen attacks various plant tissues, including roots, leaves, stems, and fruits. To manage crop diseases effectively, an integrated strategy is required, which includes appropriate cultural practices, judicious use of pesticides, and the development of resistant varieties. The creation of such resistant germplasm primarily hinges on the identification and integration of *resistance* (*R*) genes into superior cultivars via genetic crosses [1].

In their natural habitats, plants frequently suffer from attacks by pathogens, which often lead to serious disease [2,3,4]. To survive, plants utilize multiple strategies to fight against microbial attack, one of which is to avoid or minimize growth and activate the defense signal transduction. As an important class of phytohormones, auxin plays a crucial role in a wide variety of growth and development processes [5], including stem elongation, establishment of embryonic polarity [6], lateral branching of roots and shoots [7], vegetative growth [8], floral organogenesis [9], differentiation of vascular tissue [10], and seed production [11]. A series of downstream genes were induced by the response to auxin and termed as the auxin responsive genes, which include *small auxin up RNA* (*SAUR*), *Gretchen Hagen3* (*Gh3*), *Auxin/Indole-3-Acetic Acid* (*Aux/IAA*), *auxin responsive factors* (*ARF*), and *dormancy-associated gene 1* (*DRM1*)/auxin repressed protein (*ARP*) [12].

The *SAR5* gene, a member of auxin-repressed protein (ARP), was first isolated by differential screening of strawberry receptacles that were deprived of auxin [13]. The transcript level of *SAR5* was repressed by auxin, and there was a positive correlation between growth of strawberry fruit and repression of *SAR5* mRNA level [13]. Auxin-repressed protein is generally found to be a dormancy-related protein, such as PsDRM1, whose abundance of mRNA in axillary buds declines 20-fold within 6 h of decapitation and reaccumulates when buds become dormant again. A study by Park et al. showed that auxin-repressed protein RpARP negatively regulates hypocotyl elongation in *Robinia pseudoacacia*. In addition, some members of the ARP family were found to be induced and thus play vital roles in the response to biotic stress, except for the role in plant growth. For instance, ARP1/GERI1 from tobacco was reported to be a crucial mediator of the interplay between growth and plant disease resistance [14]. However, the roles of ARP in coregulation of plant growth and disease resistance still need to be unraveled, including in pepper plants.

Originally, SGT1 (suppressor of G-two allele of skp1) was defined in yeast, where it interacts with SKP1 (suppressor of kinetochore protein), a component of the Skp1Cdc53F-box protein (SCF) ubiquitin ligase complex [15]. SGT1 was also identified as an interacting partner of RAR1 (required for Mla12 resistance), which is required for multiple R (resistance) protein-mediated resistance response [16]. Further study revealed that the RAR1–SGT1 complex also interacts with two COP9 signalosome components, and the interactions among RAR1, SGT1, SCF, and signalosome subunits indicate a link between disease resistance and ubiquitination [17]. In the process of protein ubiquitination, SGT1 typically associates with the SCF complex to modulate the ubiquitination and degradation of Aux/IAA protein, a transcription activator in the auxin transduction signal pathway [18]. Despite these reports, the mechanism by which SGT1 orchestrates pepper growth and resistance to disease has not been fully investigated.

Here, we report the pepper CaARP1 as an interacting partner of CaSGT1 identified using a yeast two-hybrid screen. The interaction between CaSGT1/CaARP1 was confirmed by bimolecular fluorescence complementation (BiFC) and microscale thermophoresis (MST). We show that CaARP1 is a repressor of pepper vegetative growth and an activator of plant immunity against *P. capsici*. We also present evidence that CaARP1 positively regulates the hypersensitive response (HR)-like cell death triggered by PcINF1, an elicitin from *P. capsici*. We further implicate CaSGT1 in the effect of CaARP1 on the disease resistance of pepper plants against *P. capsici*.

## 2. Results

### 2.1. CaARP1 Interacts with CaSGT1

**Figure 1:**
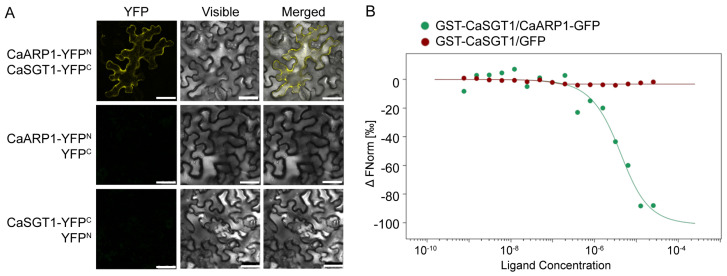
Confirmation of CaARP1/CaSGT1 interaction by bimolecular fluorescent complementary and microscale thermophoresis assays. (**A**) BiFC assay revealed the interaction between CaARP1 and CaSGT1. *Agrobacterium* cell carrying CaARP1-YFP^N^ and CaSGT1-YFP^C^ was co-infiltrated into the leaves of *N. benthamiana* using a needleless syringe. At 36–48 h post infiltration, the infiltrated leaves were sampled for the detection of the YFP signal using a confocal microscope. Bar = 25 μm. (**B**) Microscale thermophoresis experiments demonstrated that CaARP1 directly interacts with CaSGT1. Purified GST-CaSGT1 protein expressed from *E. coli* and purified CaARP1-GFP expressed from *N. benthamiana* were used in this experiment. The experiments were repeated thrice with similar results.

CaSGT1 was previously found to play an essential role in pepper immunity against *P. capsici* [19]. To further elucidate its regulatory mechanism, we initially identified CaSGT1-interacting proteins in a yeast two-hybrid screen. Among the eight CaSGT1-interacting candidates (Table S1), CaARP1 (XM_016704340.2) was chosen for further study because CaARP1 is a member of the auxin-repressed protein family, which has been implicated in plant growth and stress responses. The interaction between CaARP1 and CaSGT1 was confirmed by bimolecular fluorescence complementation (BiFC) and microscale thermophoresis assays (MST) (Figure 1). The results of the BiFC experiment revealed that CaSGT1 interacts with CaARP1 in the plasma membrane (Figure 1A), while MST results suggested the direct interaction between CaSGT1 and CaARP1 (Figure 1B).

### 2.2. The Accumulations of CaARP1 Transcript and Its Protein Product Were Up-Regulated against P. capsici 

**Figure 2:**
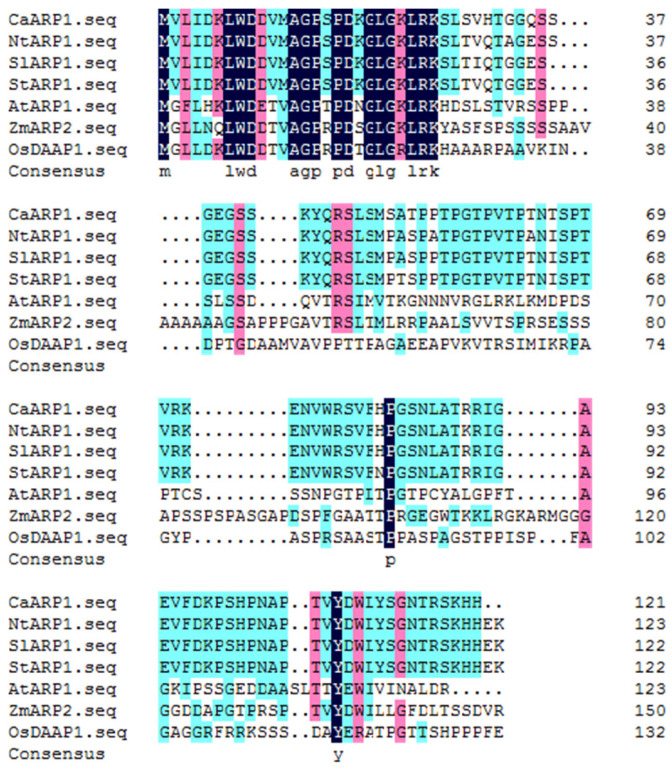
Alignments of amino acid sequence for CaARP1 protein and its orthologs from other plant species. Alignments of amino acid sequence of CaARP1 and its orthologs from *Nicotiana tabacum* (NtARP1), *Solanum lycopersicum* (SlARP1), *Solanum stenotomum* (StARP1), *Arabidopsis thaliana* (AtARP1), *Glycine max* (ZmARP2), *Oryza sativa* (OsDAAP1) and others.

The open reading frame of *CaARP1* was 375 bp and encoded a putative protein consisting of 124 amino acids and 13.49 kDa in size. Searches against National Center for Biotechnology Information (NCBI) protein databases demonstrated that CaARP1 shared high identities with auxin-repressed protein from other plant species, including 91.87% with *Nicotiana tabacum* ARP1, 93.67% with *Solanum lycopersicum*, and 91.94% with *Solanum stenotomum* (Figure 2). Conserved domain analysis revealed that CaARP1 belongs to the auxin-repressed superfamily (pfam05564), which contains several plant dormancy-associated and auxin-repressed proteins.

**Figure 3:**
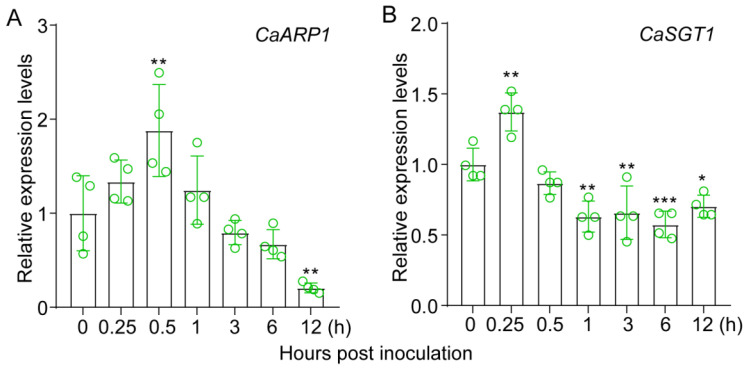
Expression profiles of pepper *CaARP1* and *CaSGT1* in response to *P. capsici* inoculation. (**A**,**B**) Quantitative RT-PCR assay showed that the transcript accumulations of *CaARP1* (**A**) and *CaSGT1* (**B**) were both up-regulated in pepper leaves challenged with *P. capsici*. Leaves of pepper plants were inoculated with the spores of *P. capsici*, and the inoculated leaves were harvested for total RNA extraction at different time-points. The transcript accumulations of *CaARP1* in pepper leaves without the inoculation of *P. capsici* were calibrated to a standardized expression level, designated as “1” for relative comparison. Green hollow dots represent four biological replicates from one experiment. The pepper *CaACTIN* was used to normalize the tested genes. Asterisks indicate significant difference as determined by one-way ANOVA with Tukey’s test (* *p* < 0.05, ** *p* < 0.01, *** *p* < 0.001). The experiments were replicated twice, yielding consistent and comparable outcomes.

To determine the response of CaARP1 in pepper plants against *P. capsici* inoculation, the expression profile of *CaARP1* in the pepper inbred line HN42 challenged with *P. capsici* was determined. The quantitative RT-PCR analysis demonstrated that the transcript accumulation of *CaARP1* started to be up-regulated at 0.25 hours post inoculation (hpi) of *P. capsici* and reached the peak at 0.5 hpi (Figure 3A). However, down-regulation of *CaARP1* transcript was detected at 12 hpi (Figure 3A). As the interacting protein of CaARP1, the transcript level of *CaSGT1* also increased at 0.25 hpi in our experimental condition (Figure 3B), which is in accordance with our previous study [19].

**Figure 4:**
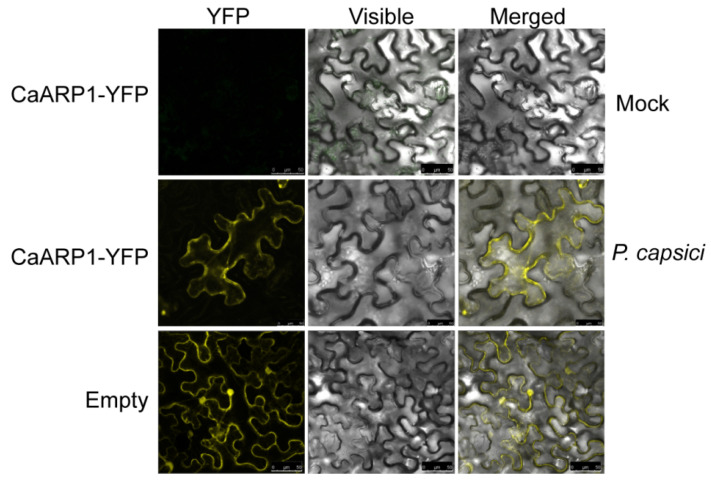
Subcellular localization of CaARP1 in leaves of *N. benthamiana* upon *P. capsici* inoculation. The leaves of *N. benthamiana* were transiently transformed with CaARP1-GFP or empty vector, and the *N. benthamiana* plants were maintained in growth chamber for 24 h, followed by the inoculation of *P. capsici* in the leaves. At 24 h post inoculation, the leaves were harvested for the detection of YFP signal. Empty: Empty vector (35S:*GFP*). Bars = 50 μm.

We further determined the effect of *P. capsici* inoculation on the accumulation of CaARP1 product in leaves of *Nicotiana benthamiana* utilizing an *Agrobacterium*-mediated transient expression experiment. To this end, *CaARP1* was fused to the coding sequences of yellow fluorescent protein (YFP) and driven by the *CaMV35S* promoter to generate 35S:*CaARP1-YFP* (CaARP1-YFP) and transient expressed in *N. benthamiana*. At 24 h post transient expression, the agroinfiltrated leaves were inoculated with *P. capsici* spores for another 24 h, followed by harvesting for YFP signal observation. Unexpectedly, no YFP signals were detected in *N. benthamiana* transiently transformed with CaARP1-YFP although driven by the *CaMV35S* promoter (Figure 4). However, the YFP signal was observed when the leaves were subjected to the inoculation of *P. capsici* spores (Figure 4). Take together, the results indicated that CaARP1 is induced in response to *P. capsici* inoculation at the transcriptional and protein level.

### 2.3. CaARP1 Knock-Down Promotes Plant Growth and Attenuates Resistance against P. capsici

**Figure 5:**
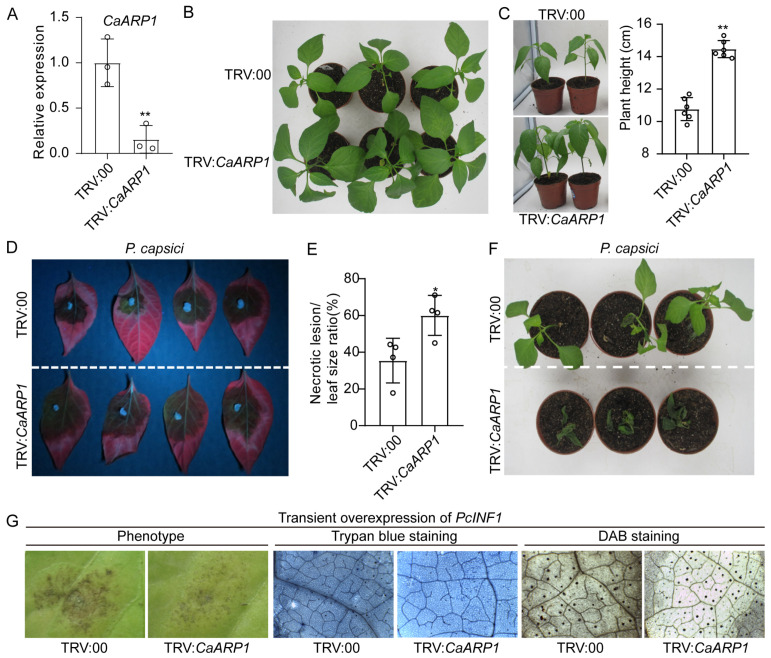
The effect of *CaARP1* silencing on the vegetative growth and disease resistance of pepper plants. (**A**) The silencing efficiency of *CaARP1* was determined by quantitative RT-PCR. The leaves of *CaARP1*-silenced and unsilenced pepper leaves at 14 days post VIGS assays were sampled for total RNA extraction. The transcript level of *CaARP1* in unsilenced pepper plants was set to a relative expression of “1”, and *CaACTIN* was used to normalize *CaARP1* expression. (**B**) Knock-down of *CaARP1* expression inhibited the vegetative growth of pepper plants. At 21 days post VIGS assays, the *CaARP1*-silenced and unsilenced pepper plants were subjected to observation for vegetative growth. (**C**) The height of pepper plants was suppressed by *CaARP1* silencing. (**D**,**E**) The disease resistance of pepper leaves against *P. capsici* was impaired by the down-regulation of *CaARP1*. The phenotype was captured at 4 dpi with *P. capsici* spores. (**F**) The inhibitive effect of *CaARP1* silencing on the disease resistance of pepper in response to *P. capsici* was also confirmed by soil-drenching inoculation. The phenotype was captured at 7 dpi. (**G**) The HR-like cell death in *CaARP1*-silenced and unsilenced pepper leaves transformed with *PcINF1*, an elicitin from *P. capsici*, confirmed by phenotype (**left**), trypan blue (**middle**) and DAB (**right**) staining. Asterisks indicate significant difference as determined by one-way ANOVA with Tukey’s test (* *p* < 0.05, ** *p* < 0.01). The experiments were replicated three times, yielding consistent and comparable outcomes.

To determine the role of CaARP1 in the vegetative growth of pepper plants, *CaARP1* was silenced in pepper plants employing the method of virus-induced gene silencing (VIGS), and whether the growth of pepper plants was altered by *CaARP1* silencing was analyzed. A quantitative RT-PCR was performed to detect the success of silencing. The relative expression of *CaARP1* in the silenced pepper plants was 30% of the plants silenced with empty vector (EV), suggesting *CaARP1* was efficiently knocked down (Figure 5A). *CaARP1*-silenced plants significantly exceeded the unsilenced pepper plants in the growth performance of germinal tops and leaves (Figure 5B). The *CaARP1*-silenced plants were almost 1.4-fold larger in height compared with the EV pepper plants (Figure 5C). Clearly, the knock-down of *CaARP1* leads to enhanced pepper growth, suggesting CaARP1 serves as a negative regulator of plant growth. Similarly, previous studies have revealed that tobacco NtARP1 negatively regulates the resistance of tobacco to several pathogens, including TMV, *Pectobacterium carotovorum* subsp. *carotovora*, and *Phytophthora parasitica* var. *nicotianae* [14]. Since the transcript level of *CaARP1* was significantly induced upon *P. capsici* inoculation, it is plausible that CaARP1 plays a role in the immune response of pepper plants against *P. capsici*. To verify this, the detached leaves of *CaARP1*-silenced pepper plants were inoculated with *P. capsici*, and the lesion spots were detected using a detective light (UV). *P. capsici* inoculation resulted in serious lesion spots in the unsilenced pepper plants, while a more serious symptom of disease was detected in the *CaARP1*-silenced pepper plants (Figure 5D,E), suggesting silencing of *CaARP1* in pepper leaves significantly attenuated its resistance to *P. capsici*. The soil drenching method was also carried out to further corroborate the positive role of CaARP1 in pepper defense against *P. capsici* attack. In line with this, a more serious symptom of leaf wilting was displayed in pepper leaves silenced with *CaARP1*, compared to the EV pepper (Figure 5F). The results above convincingly suggest the essential role of CaARP1 in disease resistance of pepper plants against attack by *P. capsici*. 

### 2.4. CaARP1 Functions in the Hypersensitive Response-like Cell Death Mediated by PcINF1

PcINF1, an elicitin derived from *P. capsici*, is a well-characterized pathogen-associated molecular pattern (PAMP) known for its ability to elicit hypersensitive response (HR)-like cell death and activate plant immunity responses. Our previous study revealed that CaSGT1 plays a vital role in the induction of HR-like cell death mediated by PcINF1 [19]. As an interactor of CaSGT1, CaARP1 was suggested to exert a role in PcINF1-mediated HR-like cell death. To this end, elicitin *PcINF1* was transiently expressed via the *Agrobacterium*-mediated expression system in pepper leaves silenced with *CaARP1* or empty vector, and HR-like cell death was evaluated. Upon transformation with *PcINF1*, the empty vector (EV) pepper plants (TRV:00) exhibited symptoms indicative of hypersensitive response (HR)-like cell death (Figure 5G, left). However, the manifestation of HR-like cell death was significantly attenuated in *CaARP1*-silenced pepper plants (Figure 5G, left), suggesting that CaARP1 plays a pivotal positive role in mediating plant cell responses in pepper leaves triggered by *PcINF1*. The inhibitive effect of *CaARP1* silencing on the HR-like cell death mediated by PcINF1 was further confirmed by trypan blue staining (Figure 5G, middle), which is an indictor of cell death. Additionally, a DAB (3,3′-diaminobenzidine) staining assay was conducted to assess the role of CaARP1 in regulating hydrogen peroxide (H_2_O_2_) accumulation in response to PcINF1 elicitation. The H_2_O_2_ accumulation generated by the transient expression of *PcINF1* was significantly suppressed by the knock-down of *CaARP1*, since a lighter dark brown color was observed in the *PcINF1*-expressed *CaARP1*-silenced pepper leaves, compared with that in the EV pepper plants (Figure 5G, right). Overall, CaARP1 positively regulates the HR-like cell death and H_2_O_2_ accumulation mediated by the elicitin PcINF1.

### 2.5. CaARP1 and CaSGT1 Positively Co-Regulate the Resistance of Pepper Plants against P. capsici

**Figure 6:**
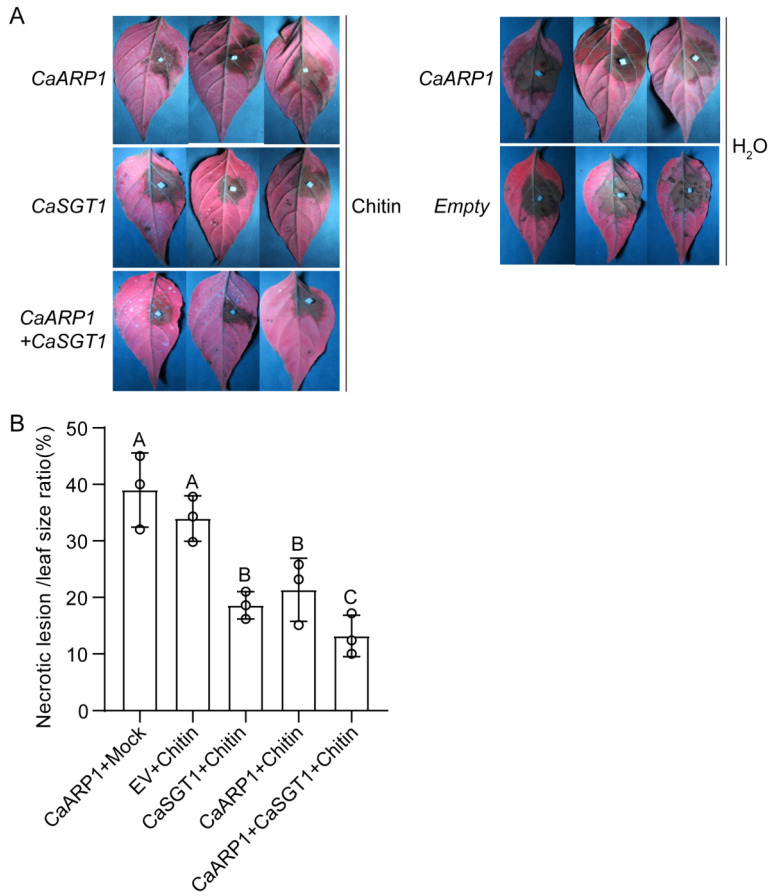
Transient expression of *CaARP1* enhanced the disease resistance of pepper leaves, which was amplified by *CaSGT1* expression. (**A**) *CaARP1* expression enhanced the disease resistance of pepper leaves challenged with *P. capsici*, and the resistance was enhanced by the transient expression of *CaSGT1*. (**B**) The necrotic lesion:leaf size ratio of pepper plants inoculated with *P. capsici*. (**A**,**B**) The leaves of pepper plants were transiently transformed with *CaARP1*, *CaSGT1*, or empty vector, and the leaves were sprayed with chitin to induce the expression of *CaARP1* and *CaSGT1*. At 24 h post spray, the leaves were inoculated with spores of *P. capsici*. Black hollow dots represent three biological replicates from one experiment. Different letters indicate significant difference as determined by one-way ANOVA with Tukey’s test (*p* < 0.05). The experiments were replicated three times, yielding consistent and comparable outcomes.

Gain-of-function experiments based on the transient expression system were further carried out to investigate the effect of *CaARP1* overexpression on the disease resistance of pepper plants challenged with *P. capsici*. Since the accumulation of CaARP1 protein driven by the *CaMV35S* promoter was particularly low in our transient expression system due to an unknown mechanism, and NtARP1 from tobacco was reported to be significantly induced by the application of chitin, a typical chitin from a fungus, we attempted to transiently express *CaARP1* (driven by *CaMV35S* promoter) in pepper leaves, followed by the application of chitin. Furthermore, the leaves transiently expressing *CaARP1* and EV were subjected to the inoculation of *P. capsici* spores, and the disease phenotype of the pepper leaves was observed using a detective light (UV light) (Figure 6). Inoculation of *P. capsici* leads to serious disease in pepper leaves transiently expressing EV, confirmed by the large lesion spot displayed in the leaves (Figure 6). However, transient expression of *CaARP1* significantly enhanced the resistance of pepper plants against *P. capsici*, since smaller sized lesion spots were detected in pepper leaves transformed with *CaARP1*, compared with those of EV (Figure 6), suggesting a positive role of CaARP1 in pepper immunity in response to *P. capsici*. As an interactor with CaARP1, CaSGT1 serves a positive role in modulating the immune response of pepper leaves against attack by *P. capsici*, since the lesion spots in *P. capsici*-inoculated pepper leaves transiently expressing *CaSGT1* were smaller than those on leaves from the EV plants (Figure 6). Of note, co-expression of *CaARP1* and *CaSGT1* enhanced the resistance of pepper leaves against *P. capsici* to a greater extent, compared to the individual expression of *CaARP1* or *CaSGT1*, since the smallest lesion spots were observed in pepper leaves co-transformed with *CaARP1* and *CaSGT1* among all the tested groups (Figure 6). Taken together, these results suggest CaARP1 serves as a positive regulator in the defense of pepper plants against *P. capsici* in a manner associated with CaSGT1. 

## 3. Discussion

To identify additional factors required for CaSGT1-mediated pepper immunity against *P. capsici*, interacting proteins of CaSGT1 were isolated using a yeast two-hybrid system. This analysis identified several CaSGT1-interacting proteins (Table S1), including CaARP1, which is described in the present study. CaARP1 belongs to the auxin-repressed protein family, which is involved in the regulation of plant growth. We provide biochemical data demonstrating that CaARP1 is indeed an interacting protein of pepper CaSGT1. Our data further imply that CaSGT1 and CaARP1 may collaborate in orchestrating the intricate crosstalk between the growth and disease resistance in pepper plants.

As auxin-repressed proteins, the ARP proteins were reported to primarily participate in the regulation of plant growth by engaging in the auxin signaling pathway [14,20,21,22]. Our experiments indicated that knock-down of *CaARP1* inhibited the vegetative growth of pepper plants (Figure 5), suggesting the negative role of CaAPR1 in pepper growth. Despite their involvement in plant growth, ARP proteins were reported to be induced by pathogen invasion and participate in the modulation of immune response [23,24], although there had been a scarcity of information regarding the role of auxin signaling in disease resistance until recent years. Modulation of the signaling pathway, including that of auxin, is a well-established strategy that is utilized by the plant to balance growth and innate immunity [14]. However, the molecular mechanism by which the auxin signaling pathway is regulated remains largely unknown. 

It is well-established that the transcript expression of many genes involved in the defense reaction against pathogens is generally induced by the invasion of pathogens. For instance, another member (NCBI LOCUS: AF082729) of the ARP family from pepper was also found to be induced by the infection of *Xanthomonas campestris* pv. *vesicatoria* [23,24]. In the present study, we found the transcript level of *CaARP1* to be induced by *P. capsici* attack (Figure 2 and Figure 3) and the silencing of *CaARP1* to attenuate the disease resistance of pepper plant (Figure 5), suggesting a positive contribution of this auxin-repressed protein to resistance against *P. capsici*.

Of note, we failed to observe the fluorescent signal of CaARP1-YFP fusion protein expressed via the *Agrobacterium*-mediated transient expression even though driven by the constitutive promoter *CaMV35S* (Figure 4), probably due to the low expression level of CaARP1-YFP product or the degradation of CaARP1-YFP protein. We hypothesize that when the *CaARP1* gene is transiently expressed in leaves, plants may impede the accumulation of the CaARP1 protein through mechanisms such as mRNA degradation or post-translational protein degradation from affecting plant growth, since ARP protein is generally reported to be a repressor protein of plant growth. However, we were able to observe the CaARP1-YFP protein using a confocal microscope, when the leaves were challenged with *P. capsici* (Figure 4). It seems that *P. capsici* inoculation induces the accumulation of CaARP1 protein, which might repress plant growth and activate plant immunity. Indeed, our data revealed that the silencing of *CaARP1* promotes the vegetative growth of pepper plants but inhibits their resistance to *P. capsici*, suggesting a dual role of CaARP1 in the regulation of vegetative growth and immune response in pepper plants. Similarly, auxin-repressed protein ARP1 from tobacco was reported to regulate growth and disease resistance in tobacco [14]. We hypothesize that CaARP1 serves as a pivotal component mediating the auxin signaling cascade, thereby modulating the intricate interplay between plant growth and disease resistance of pepper.

INF1, an elicitin from *Phytophthora* and *Pythium* spp., was determined to be a typical PAMP that induces the hypersensitive response (HR)-like cell death in many Solanaceae plants, including *Nicotiana*, *Brasscia* spp. and pepper plants. Many components were reported to participate in PAMP-triggered immunity mediated by oomycete INF1, including SGT1 [25,26]. As an interactor with pepper CaSGT1, CaARP1 was hypothesized to function in the immunity triggered by INF1. Our previous study indicated that PcINF1 from *P. capsici* induced HR-like cell death in pepper leaves, as confirmed by agroinfiltration or infiltration of recombinant PcINF1 protein expressed in *Escherichia coli* [27]. Herein, our study demonstrates that the HR-like cell death triggered by PcINF1 was significantly impaired by the knock-down of *CaARP1* (Figure 5), suggesting a positive role of CaARP1 in pepper immunity mediated by the response to *P. capsici*. Consistent with this, a study by Zhao et al. revealed that ARP1 regulates tobacco’s response to other PAMPs, including flg22 and chitin, and thus is involved in the induction of PTI response [14]. Accordingly, the role of auxin-repressed protein in the regulation of PTI response seems to be conserved in Solanaceae plants, at least including tobacco and pepper. Given the fact that CaSGT1 interacts with CaARP1 (Figure 1), we hypothesize that CaARP1 may participate in the defense response of pepper plants against *P. capsici* in a manner associated with CaSGT1. To this end, our assays showed that overexpression of *CaARP1* in pepper leaves significantly enhanced its disease resistance against *P. capsici*, which was amplified by the co-expression of *CaSGT1* (Figure 6), suggesting the CaSGT1-CaARP1 module regulates pepper immunity in response to *P. capsici* in a collaborative way. Previous study showed that SGT1 associates with SCF complexes and facilitates SCF assembly [18]; we speculate that as an interactor with CaARP1, CaSGT1 might contribute to the stability of CaARP1 protein, thus participating in maintaining the balance between plant growth and immunity mediated by CaARP1.

Collectively, our study revealed that CaARP1 serves a dual function in the pepper plant, exhibiting negative regulation of the vegetative growth and positive regulation of disease resistance against *P. capsici* in a manner associated with CaSGT1. However, by which mechanism the CaARP1-CaSGT1 module modulates the tradeoff between plant growth and immunity remains to be further investigated.

## 4. Materials and Methods

### 4.1. Plant Materials and Pathogen Inoculation

In this study, seeds of *Capsicum annuum* cultivar 130 and *Nicotiana benthamiana*, sourced from a pepper breeding center at Fujian Agriculture and Forestry University, were employed. Following a stratification period at 4 °C for 2 days, all seeds were germinated in sterilized deionized water (ddH_2_O). After 7 days of growth, the seedlings were transferred to a plastic pot (8 cm diameter) containing a soil mixture and maintained in a greenhouse under conditions of 25 °C, 60% relative humidity, and a 16 h light/8 h dark photoperiod. Plants aged 4–6 weeks were then subjected to pathogen inoculation. Prior to inoculation, the roots of pepper plants were damaged by inserting a knife into the soil to a depth of 5 cm a total of three times. The soil was subsequently irrigated with *P. capsici* suspended in sterilized ddH_2_O. Post-inoculation, the plants were grown in a growth chamber set at 28 °C, 80% relative humidity, and a 16 h light/8 h dark photoperiod.

### 4.2. P. capsici Infection Assay

The *P. capsici* strain JX-1 used in this study was recovered on 10% V8 medium, and then placed in a constant temperature incubator at 25 °C for dark culture for 5–7 days until the whole Petri dish was covered with white colonies that could be used for subsequent experiments. For the purpose of mycelium inoculation onto *Nicotiana benthamiana* and pepper plants, the leaves underwent an incubation period of 24 to 30 h with mycelial plugs. For the zoospore infection assays, the mycelial plugs were subjected to an incubation period of 3 days in liquid V8 medium, followed by three consecutive washes with sterile water to ensure aseptic conditions. To release the zoospores, the cultures were incubated at 4 °C for 0.5–1 h, and then placed in an incubator at 25 °C for 0.5–1 h to collect spore water. The concentration of zoospore suspension was adjusted with sterile water to 1 × 10^4^/mL for further inoculation assay. The inoculated leaves were photographed under ultraviolet (UV) light at 56 to 72 h post-inoculation, and the resulting lesion areas were subsequently quantified utilizing the Image J 1 software tool.

### 4.3. Plasmid Construction and Recombinant Proteins

The coding sequences for *CaSGT1* were amplified from pepper cDNA to create recombinant protein expression vectors. These amplified fragments were then inserted into the appropriate vectors (GST-tagged). The resulting fusion recombinant proteins were expressed in *Escherichia coli* DE3 strains. These bacteria were cultivated in liquid Luria-Bertani (LB) medium at a temperature of 37 °C until they reached an optical density at 600 nm (OD600) between 1.0 and 1.5. The recombinant glutathione S-transferase (GST) proteins were elicited via the application of 0.2 mM isopropyl β-D-1-thiogalactopyranoside (IPTG) and subsequently underwent purification utilizing glutathione sepharose (GE, Schenectady, NY, USA).

### 4.4. Subcellular Localization

*Agrobacterium* GV3101 cells that contain *CaARP1*-YFP fusion constructs were cultivated in YEP medium. Following cultivation, the bacterial cells were harvested through centrifugation, resuspended in an infiltration medium composed of 10 mM MgCl_2_, 10 mM MES at a pH of 5.7, and 200 μM acetosyringone, and then adjusted to an optical density at 595 nm (OD595) ranging between 0.6 and 0.8. These suspensions were subsequently infiltrated into the leaves of 6-week-old *N. benthamiana* plants via vacuum infiltration using a needleless syringe. The plants were then maintained in a controlled growth chamber environment. Detection of fluorescent signal was performed on the infiltrated *N. benthamiana* leaves at 36 to 48 h post-infiltration, employing a confocal laser scanning microscope SP8 (Leica, Wetzlar, Germany). The excitation and emission wavelengths for GFP fluorescence were set at 488 nm and between 510 and 520 nm, respectively.

### 4.5. Yeast Two-Hybrid Assays

The isolation of total RNA from the leaves of plants aged 4 to 5 weeks was performed using TRIzol reagent (Invitrogen, Waltham, MA, USA). Subsequently, the mRNA fraction was then purified using mRNA purification kits. For the implementation of Y2H, the GAL4 system was employed, adhering strictly to the manufacturer’s instructions (Invitrogen). For the Y2H screen, the open reading frame of *CaSGT1* was amplified and cloned into the Gateway satellite vector pDONR201 (Invitrogen) to generate pDONR201-CaSGT1 using a BP reaction. The generated satellite vector was then transferred into the destination vector pDEST32 (Invitrogen) using an LR reaction. The constructs were subsequently transformed into the yeast strain MaV203. Positive clones were selected after growth on synthetic dropout (SD) medium without leucine (SD/-Leu). The large-scale cDNA library was transformed into the MaV203 strain, which contained *CaSGT1* and allowed growth on SD/−Leu//-/−Trp//-/−-His/+appropriate concentrations of 3-amino-1,2,4-triazole (3AT).

### 4.6. Bimolecular Fluorescence Complementation

The coding sequences for *CaSGT1* and *CaARP1* were amplified from pepper cDNA utilizing attB adaptor-linked primers and inserted into the bimolecular fluorescence complementation (BiFC) vectors pDEST-SCYNE and pDEST-SCYCE, resulting in the generation of CaARP1-SCYNE and CaSGT1-SCYCE constructs, respectively. *Agrobacterium tumefaciens* strain GV3101, carrying CaARP1-SCYNE and CaSGT1-SCYCE, was co-infiltrated into the leaves of *N. benthamiana*. The infiltrated plants were then cultivated in a greenhouse environment. Approximately 36 to 48 h post-infiltration, the leaves subjected to agroinfiltration were harvested, and a confocal laser scanning microscope was employed to detect fluorescence signals. The excitation and emission wavelengths were set at 513 nm and between 525 and 535 nm, respectively.

### 4.7. Quantitative RT-PCR Analysis

Quantitative reverse transcription polymerase chain reaction (qRT-PCR) was conducted as delineated in the study by Liu et al. [27]. In summary, total RNA was isolated from pepper using the TRIzol reagent. The genomic DNA contaminants present in the extracted RNA samples were eliminated through digestion with DNase I. The synthesis of first strand complementary DNA (cDNA) was accomplished using a cDNA synthesis kit (Vazyme, Nanjing, China), following the stipulations provided by the manufacturer. The quantitative PCR (qPCR) was performed using a Bio-rad real-time PCR system (Bio-rad, Hercules, CA, USA) and the AceQ qPCR SYBR Green Master Mix (Vazyme, Nanjing, China). The pepper *CaACTIN* gene served as the internal reference standard. The oligonucleotide primers employed in this research are listed in Table S2. 

### 4.8. Agrobacterium-Mediated Transient Expression

The method for *Agrobacterium*-mediated transient expression was carried out as outlined, with minor adjustments, as delineated by Choi et al. [28]. The *Agrobacterium tumefaciens* strain GV3101, carrying the relevant constructs, was cultivated overnight in Luria-Bertani (LB) liquid medium supplemented with 75 μg/mL rifampicin and 75 μg/mL kanamycin. Subsequently, the bacterial cells were harvested through centrifugation and then resuspended in an infiltration medium consisting of 10 mM MgCl_2_, 10 mM MES, and 200 μM acetosyringone (at a pH of 5.7). This bacterial cell suspension was introduced into the leaves of either pepper or *N. benthamiana* using a needleless syringe. Approximately 36 to 48 hours post-infiltration (hpi), the leaves that had been subjected to agroinfiltration were collected for subsequent analytical procedures.

### 4.9. VIGS Assay

The virus-induced gene silencing (VIGS) assay was conducted with minor alterations as delineated by Liu et al. [29]. Specific fragments of the *CaARP1* gene were amplified utilizing gene-specific primers (listed in Table S2) and subsequently inserted into the VIGS vector TRV2, resulting in the formation of TRV2:*CaARP1*. The uniqueness of these fragments was verified through BLASTN searches on the pepper genome database (http://passport.pepper.snu.ac.kr/?t=PGENOME, accessed on 1 July 2017). *Agrobacterium* cells, transformed with TRV1 and TRV2:*CaARP1*, were cultivated, collected, and then resuspended in an infiltration medium composed of 10 mM MgCl_2_, 10 mM MES at a pH of 5.7, and 200 μM acetosyringone. *Agrobacterium* cultures containing TRV1 and TRV2 were admixed in a 1:1 ratio, incubated at 25 °C for a duration of 3–4 h, and thereafter infiltrated into the cotyledons of pepper plants at the four-leaf stage. Both silenced and control pepper plants were employed in subsequent experiments 3–4 weeks following agroinfiltration. Pepper plants that underwent agroinfiltration with TRV1 and TRV2 served as the negative control group.

### 4.10. Statistical Analyses

The variances between the two groups were denoted by a single asterisk (*) for statistical significance at the *p* < 0.05 level, a double asterisk (**) for greater significance at the *p* < 0.01 level, and triple asterisks (***) for extreme significance at the *p* < 0.001 level, as determined by one-way ANOVA with Tukey’s test. Differences among multiple groups were indicated by distinct letters, signifying a significance level of *p* < 0.01, as determined using one-way ANOVA with Tukey’s test.

## Data Availability

The raw data supporting the conclusions of this article will be made available by the authors on request.

## Supplementary Materials

The following supporting information can be downloaded at: https://www.mdpi.com/article/10.3390/plants13202849/s1, Table S1: Candidate interacting-partners (CIP) for CaSGT1 as identified through a yeast two-hybrid assay; Table S2: Oligonucleotides used in this study.
